# Supplementary material for: The Diverse Transformer (Trf) Protein Family in the Sea Urchin Paracentrotus lividus Acts through a Collaboration between Cellular and Humoral Immune Effector Arms
Source: Int J Mol Sci. 2021 Jun 22;22(13):6639. doi: 10.3390/ijms22136639 (PMC8268236; doi:10.3390/ijms22136639)
Supplement: Supplementary file 1 [file ijms-22-06639-s001.zip › ijms-1256088-supplementary.pdf]

Diverse Transformers (Trf) protein family in the sea urchin *Paracentrotus lividus* act through collaboration between cellular and humoral immune effector arms.

Iryna Yakovenko[1], Asaf Donnyo[1], Or Ioscovich[1], Benyamin Rosental[2], Matan Oren[1*]

Affiliations:

[1] Department of Molecular Biology, Ariel University, Science Park, Ariel 40700, Israel

[2] The Shraga Segal Department of Microbiology, Immunology and Genetics, Regenerative Medicine, and Stem Cell Research Center, Ben-Gurion University of the Negev, Beer Sheva 84105, Israel

* Correspondence to: matanor@ariel.ac.il

Supplementary material

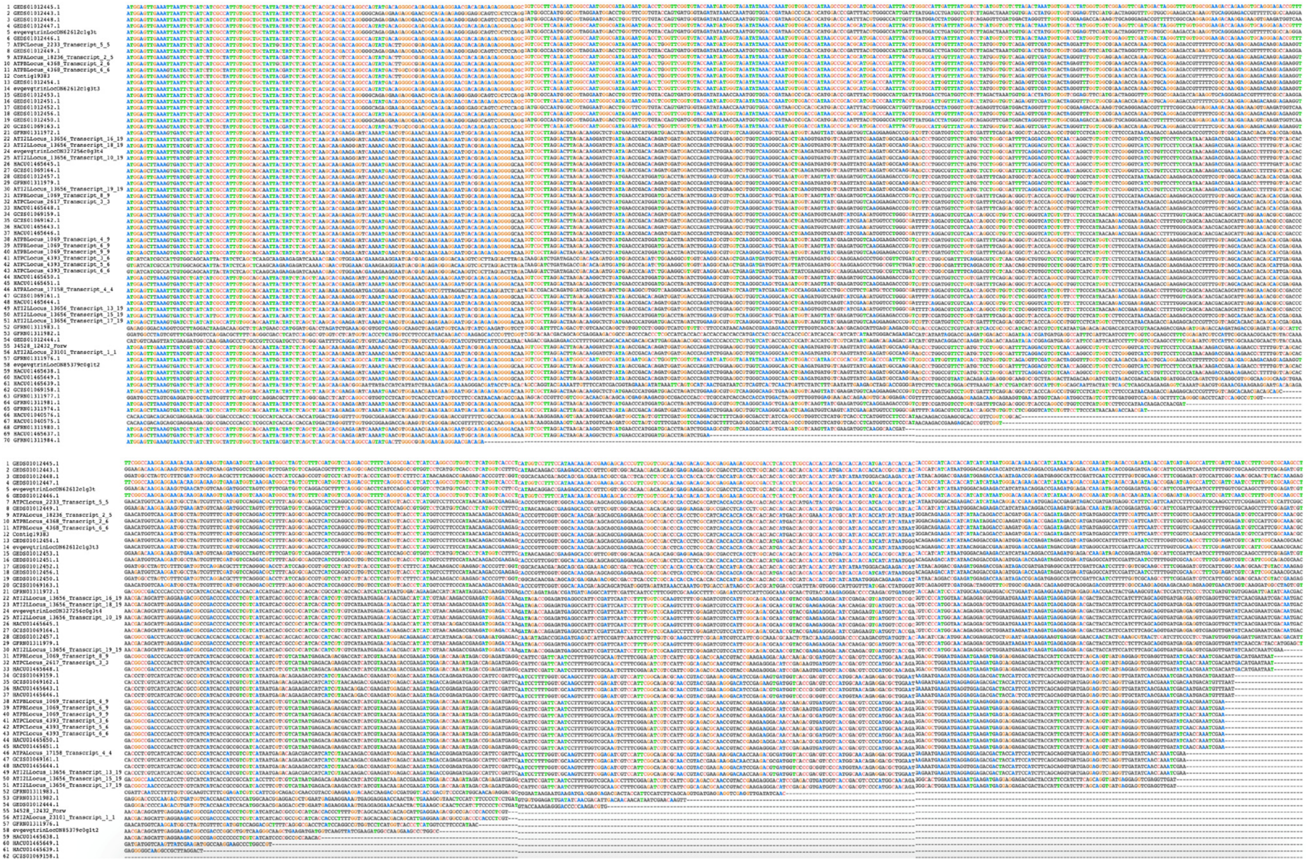

Figure S1 Unique Trf sequences obtained from available databases.

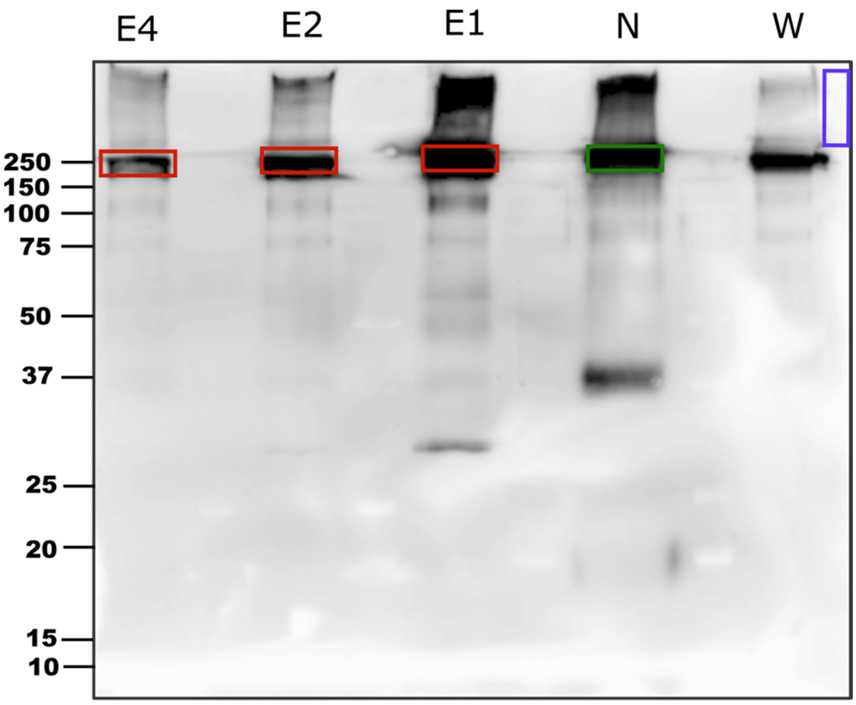

Figure S2 Western blot of the gel, identical to which was used for the mass spectrometry. E1, E2, E4 – elution fractions, N – untreated native whole coelomic fluid protein, W – wash fraction. Inside boxes are the bands that were cut from PAGE gel. In red – elution fractions, in green – native protein from the same animal, in blue – the background control that was cut from the side of the gel.

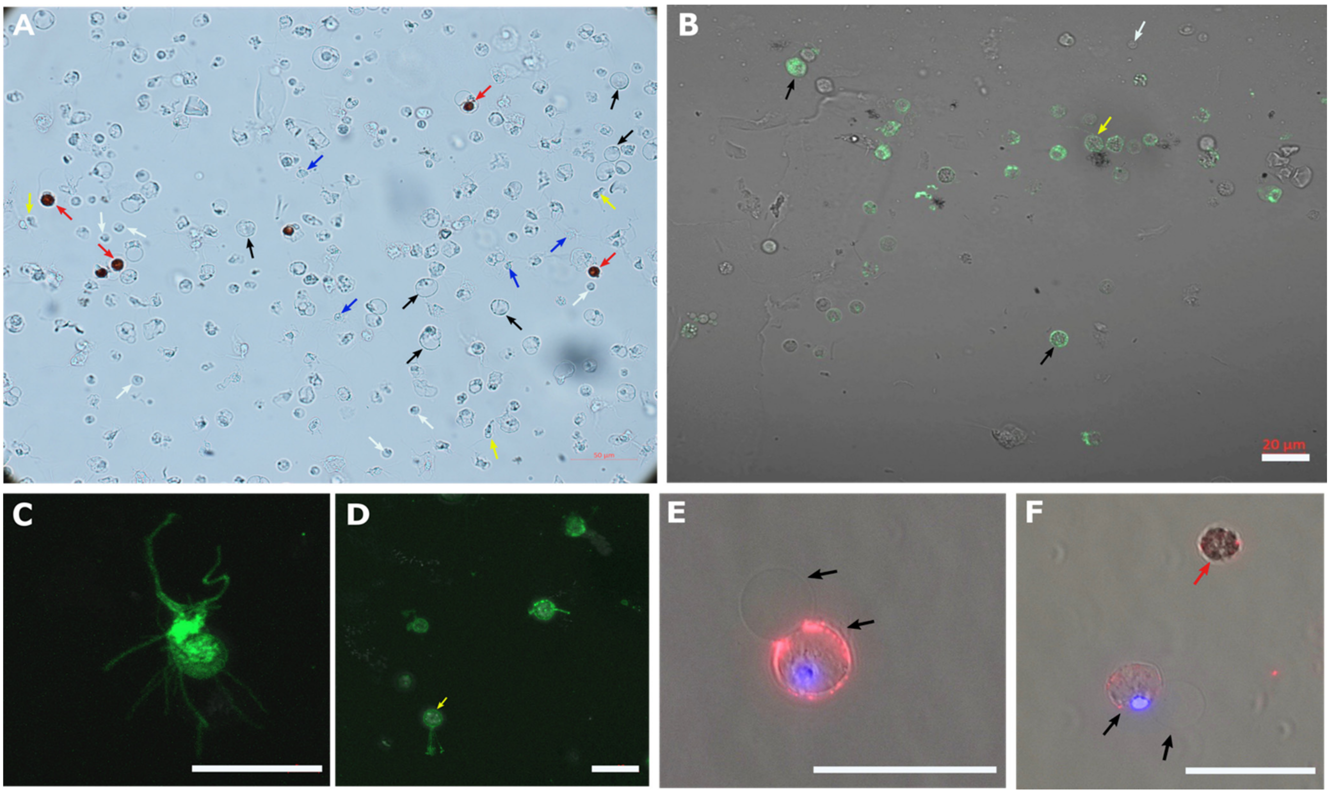

Figure S3. ***P. lividus*** **coelomocyte types and sorted Trf-positive coelomocytes. A.** *P. lividus* unsorted coelomocytes in whole CF shown in a bright field. **B.** Sorted Trf-positive cells - petaloid phagocytes and a vibratile cell labeling is clearly seen, other coelomocyte types have a weaker signal. **C.** Trf protein expression on the cell surface and filopodia of a filopodial phagocyte (confocal microscopy) **D.** Trf-positive *P. lividus* vibratile cell (yellow arrow) (confocal microscopy). **E and F.** Membranal interactions of Trf-positive and Trf-negative petaloid phagocytes (black arrows). Scale bars = 20 µm. Blue arrows - filopodial phagocytes. Red arrows- red

spherule cells. Yellow arrows - vibratile cells. Green fluorophore — 2nd Ab AlexaFluor 488. Red fluorophore - 2nd Ab AlexaFluor 567. Blue is DAPI.

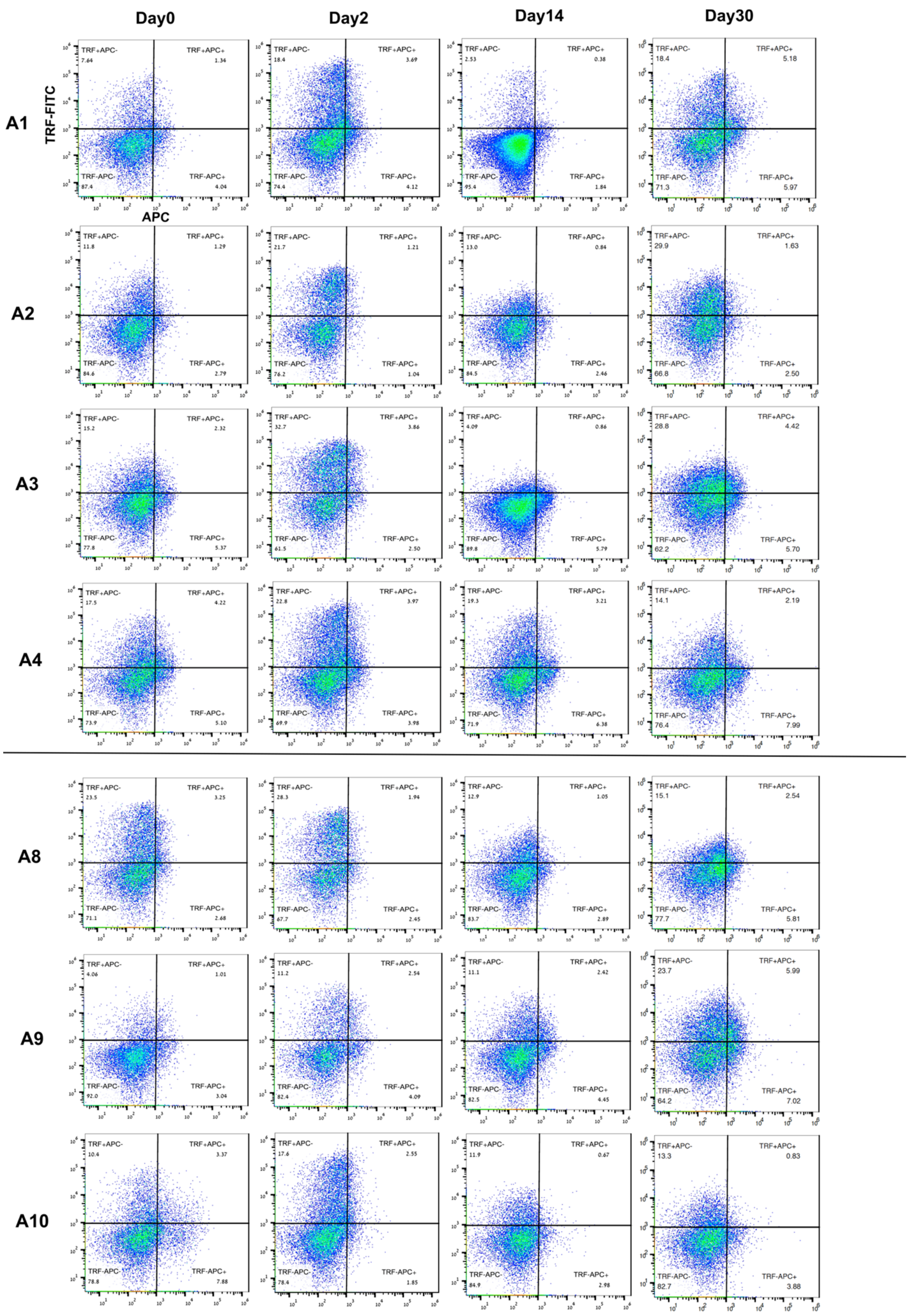

Figure S4 Trf-positive cell population change over time after injection of either *V. penaeicida* or Acf. A1-A4 – animals challenged with heat-killed *V. penaeicida*, A8-A10 - control animals injected with

sterile aCF. Y-axis Trf labeled with AF488, X-axis autofluorescence in APC channel indicates red spherule cells.

Table S1 accessions of the Trf sequences used to build the phylogenetic tree.

H. erythrogramma*		***S. purpuratus*s**		*P. lividus***	
**JX245053.1**	**HE001**	**KJ408453.1**	SP001	HACU01465650.1	PL001
JQ780274.1	HE002	KJ408451.1	SP002	HACU01465648.1	PL002
JX245045.1	HE003	KJ408450.1	SP003	HACU01465646.1	PL003
JQ780273.1	HE004	KJ408449.1	SP004	HACU01465645.1	PL004
JX245024.1	HE005	EF607775.1	SP005	HACU01465644.1	PL005
JX245021.1	HE006	EF607759.1	SP006	HACU01465643.1	PL006
JQ780304.1	HE007	EF607755.1	SP007	GFRN01311981.1	PL007
JX245020.1	HE008	EF607753.1	SP008	GFRN01311979.1	PL008
JQ780312.1	HE009	EF607742.1	SP009	GFRN01311974.1	PL009
JQ780311.1	HE010	EF607719.1	SP010	GFRN01311972.1	PL010
JQ780305.1	HE011	EF607717.1	SP011	GEDS01012457.1	PL011
JQ780303.1	HE012	EF607713.1	SP012	GEDS01012456.1	PL012
JQ780287.1	HE013	EF066272.1	SP013	GEDS01012454.1	PL013
JQ780284.1	HE014	EF066267.1	SP014	GEDS01012453.1	PL014
JQ780276.1	HE015	EF066259.1	SP015	GEDS01012452.1	PL015
JQ780275.1	HE016	EF066253.1	SP016	GEDS01012451.1	PL016
JQ780271.1	HE017	EF066219.1	SP017	GEDS01012450.1	PL017
JQ780270.1	HE018	EF066159.1	SP018	GEDS01012448.1	PL018
JQ780269.1	HE019	EF065743.1	SP019	GEDS01012447.1	PL019
JQ780268.1	HE020	EF065742.1	SP020	GEDS01012446.1	PL020
JQ780266.1	HE021	EF065733.1	SP021	GEDS01012445.1	PL021
JQ780265.1	HE022	DQ183180.1	SP022	GEDS01012443.1	PL022
JQ780262.1	HE023	DQ183182.1	SP023	GCZS01069164.1	PL023
JQ780244.1	HE024	DQ183168.1	SP024	GCZS01069162.1	PL024
JQ780233.1	HE025	DQ183167.1	SP025	GCZS01069159.1	PL025

* *P. lividus*, *S. purpuratus* and *H. erythrogramma* Trf sequence accession numbers from NCBI protein database
***P. lividus* sequences from TSA NCBI databases (HACU01, GCZS01, GEDS01, GFRN01)
